# Pathology of Sea Sickness

**Published:** 1889-03-02

**Authors:** 


### Medical Aids and Appliances.

### PATHOLOGY OF SEA-SICKNESS.

Dr. J. A. Irwin, late hon. physician to the Manchester Southern Hospital for Women and Children, recently published a pamphlet entitled "Preliminary Observations on the Pathology of Sea-sickness." The author states that "our bodies are endowed with what may be termed a supplementary special sense, quite independent of, but at the same time in the closest alliance with our other special senses, the function of which is to determine the position of the head in space, and to govern and direct the æsthetiko-kinetic mechanism by which is maintained the equilibrium of the body." Sea-sickness, it is stated, is essentially a disturbance of this function. The author has preparing for publication a full treatise on the subject, which, however, if published, we have not seen, and therefore, instead of criticising the pamphlet before us, we await the appearance of the large volume.

